# Effects of heat-moisture treatment on the thermal, functional properties and composition of cereal, legume and tuber starches—a review

**DOI:** 10.1007/s13197-020-04520-4

**Published:** 2020-07-12

**Authors:** Vhulenda Melinda Mathobo, Henry Silungwe, Shonisani Eugenia Ramashia, Tonna Ashim Anyasi

**Affiliations:** 1 Department of Food Science and Technology, School of Agriculture, University of Venda, Private Bag X5050, Thohoyandou, Limpopo Province 0950 South Africa; 2 Department of Food Science and Technology, Cape Peninsula University of Technology, P.O. Box 1906, Bellville, 7537 South Africa

**Keywords:** Starch, Hydrothermal modification, Heat-moisture treatment, Thermal properties, Functional properties, Starch modification

## Abstract

Several methods are currently employed in the modification of starch obtained from different botanical sources. Starch in its native form is limited in application due to retrogradation, syneresis, inability to withstand shear stress as well as its unstable nature at varying temperatures and pH environment. Modification of starch is therefore needed to enhance its food and industrial application. A primary and safe means of modifying starch for food and industrial use is through hydrothermal methods which involves heat-moisture treatment and annealing. Heat-moisture treatment (HMT) is a physical modification technique that improves the functional and physicochemical properties of starch without changing its molecular composition. Upon modification through HMT, starches from cereals, legumes and tuber crops serve as important ingredients in diverse food, pharmaceutical and industrial processes. Although changes in starch initiated by HMT have been studied in starches of different plant origin, this work further provides insight on the composition, thermal and functional properties of heat-moisture treated starch obtained from cereals, legumes and tuber crops.

## Introduction

Starch is a polysaccharide that is composed of two α-glucan polymerized molecules: amylose (AM), the minor component and amylopectin (AP) which is the major component in most starches (Vamadevan and Bertoft 2015; Barbi et al. 2018). Amylose is a linear chain of D-glucose units with an α-(1,4) linkage while AP comprise branches of D-glucose from an α-(1,4) linkage at the branches, to α-(1,6) linkage at the branch points (Pratiwi et al. 2018). Starch is present in plant tissues in granular form, appearing in different granular shapes and sizes where it serves as the major storage carbohydrate reserve (Hoover 2010). The glucans that make up the starch granules are deposited to yield semi crystalline starch granules having varying shapes, size and conformation (Chibbar et al. 2010).

**Fig. 1:**
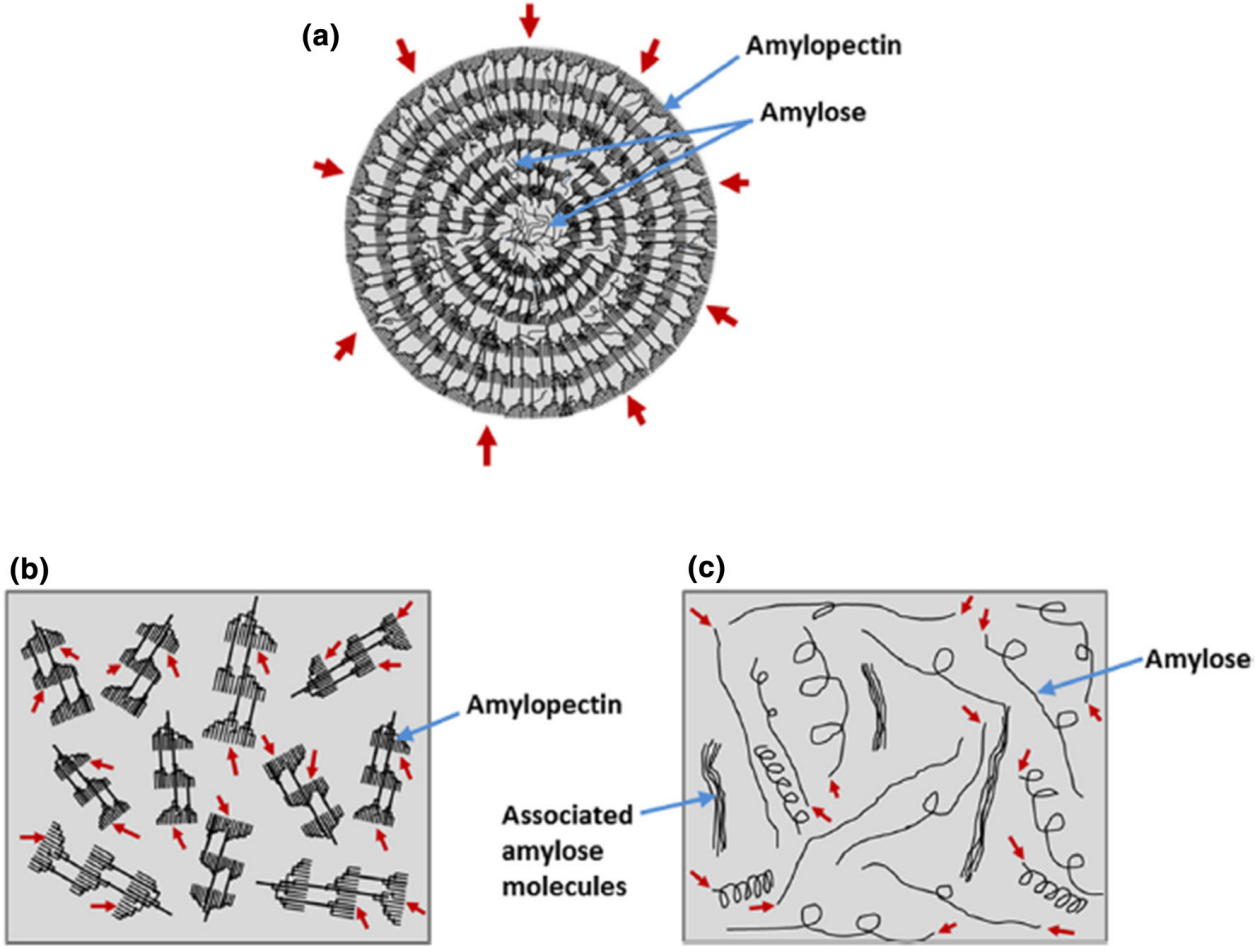
Reaction pattern of amylases on amylose and amylopectin. **a** semicrystalline structure of native granule; **b** dispersed amylopectin molecules; and **c** dispersed amylose molecules. Arrows shows access points of amylases towards the negative granules (**a**), isolated amylopectin (**b**) and amylose (**c**) of starches. *Source*: Naguleswaran et al. (2014)

The internal structure of starch granules is made up of concentric alternating amorphous and semi crystalline growth rings emanating from the hilum of the granule (Fig. 1). These amorphous rings are made up of AM and AP in a chaotic conformation, while the semi crystalline rings are shaped by a lamellar structure of alternating crystalline and amorphous regions with a repeat distance of 9–11 nm. Within these lamellae, the crystalline regions are said to be produced by AP chains crammed into a crystalline lattice, even though the amorphous regions contain the AP branching points and AM and AP molecules in a disordered manner. The crystalline regions of lamellae are mainly formed by double helices of AP side chains packed into different polymorphic A-, B- and C-type forms (Maaran et al. 2014).

Starch in its application, significantly affects the textural properties of food and holds countless industrial applications as thickeners, colloidal stabilizers, gel improvers as well as its use as bulking and water retention agent (Singh et al. 2003). Starch also brings about texture modification when used as an additive in foods (Sun et al. 2015). Native starches obtained from different botanical sources are restricted in their use due to inherent characteristic in these starches such as cohesive texture, heat and shear sensitivity, lack of clarity, opacity, low viscosity and precipitation during storage (Sharma et al. 2015). Native starch is also limited in its application due to its distinctive nature: insoluble in water, high degree of retrogradation, low stability to freeze thawing, syneresis and formation of non-stable paste and gel (Garrido et al. 2014; Beninca et al. 2013; Andrade et al. 2014; Ashogbon 2014). Starch is therefore modified to enhance its properties and use during processing (Alimi et al. 2016). Modification of starch results in significant value addition, enhancement of starch application and provides the basis for the development of various food products with enhanced functional and nutritional properties (Sharma et al. 2015). The properties of starches obtained from different botanical sources can thus be improved by the regulated application of heat and moisture, thereby resulting in an environmentally friendly and safe physical modification of the starches (Anderson and Guraya 2005).

## Cereals, legumes and tuber starch

Starch, a major carbohydrate storage of cereals (Shevkani et al. 2017), legume seeds (Klamczynska et al. 2001; Wani et al. 2016) and tubers (Hermiati et al. 2012) is also the most important source of energy in these crops. Native starch is a very essential carbohydrate for human nutrition and energy supply, hence, its importance in health cannot be overlooked. Despite the functionalities of native starch, unmodified starches are restricted in their use as a result of existing limitations. However, native starches obtained from cereals, legumes, roots and tubers can be modified to obtain desirable and acceptable qualities through different starch modification techniques. Such modification methods include the physical, chemical and enzymatic modification methods through which the physicochemical characteristics of starch can be altered and improved on. Extensive studies have therefore been conducted on the impact of the physical and chemical modification methods on the functional and physicochemical characteristics of starches obtained from different plant sources (Adebowale and Lawal 2002, 2003; Chandla et al. 2017; Liu et al. 2016; Gunaratne and Hoover 2002; Lee et al. 2005; Sarkar 2015).

### Composition of cereals, legumes and tuber starch

Starch granules consist of two major polysaccharides (AM and AP) which account for approximately 98–99% (dry weight) (Sulaiman 2011). Starches with extended AM content are reported to contain intermediate constituents having structures intermediate to those of AP and AM. Vamadevan and Bertoft (2015) further showed that starch granules consist of minute amounts of proteins, fatty acids and minerals which affects the properties of the starch. The lipid concentration of native starches correlates highly with the concentration of AM, as an increase in AM content leads to an increase in the amount of lipid present (Copeland et al. 2009). In the works of various authors, AM, protein, lipid, ash and moisture contents of starches from cereals, legumes, root and tuber sources where seen to vary (Table 1). The dissimilarities in the composition of the starches from different botanical sources can be attributed to cultivar differences, estimation procedure and environmental conditions (Ashogbon 2014).

**Table 1:** Chemical composition of native starch from different botanical sources

Starch source	Ash (%)	Lipid (%)	Moisture (%)	Amylose (%)	Protein (%)	References
Chickpea	0.05–0.06	0.29–0.50	8.78–11.45	23.00–33.81	0.03	Wani et al. (2016), Huang et al. (2008)
Kidney bean	0.25–0.50	0.10	10.88–11.00	42.96–49.28	0.08–0.10	Wani et al. (2016)
Corn		0.70–0.80		28.00	0.35	Yuryev et al. (2002)
Wheat		0.33–0.48		25.3–26.8	0.44–0.63	Shevkani et al. (2017)
Lentil	0.03–0.25	0.09–0.40	8.90–9.40	22.10–33.90	0.03–0.09	Wani et al. (2016)
Green gram	0.25–0.50	0.10–0.32	10.63–11.96	34.47–45.30	0.02–0.05	Wani et al. (2016), Andrabi et al. (2015)
Moth bean	0.62	0.87	9.48	26.42	0.06	Wani et al. (2016)
Smooth pea	0.62	0.04	3.12	22.00–27.90	0.03–0.04	Wani et al. (2016)
Black bean	0.14	0.20–0.50	16.00	27.20–39.30	0.04–0.07	Wani et al. (2016)
Pinto bean	0.06	0.12	16.00	31.30–37.40	0.05–0.07	Wani et al. (2016)
Durum wheat		0.39–0.47		26.20–30.20	0.44–0.57	Shevkani et al. (2017)
Potato	0.25	0.05–0.10		25.20–29.10	0.30–0.34	Gunaratne and Hoover (2002)
Faba bean	0.03	0.08–40.00		17.00–42.00	0.33–0.43	Wani et al. (2016), Gunasekera et al. (1999)
Cowpea		0.20–1.33		25.80–33.00	0.06–0.09	Wani et al. (2016), Huang et al. (2008), Gunasekera et al. (1999)
Bambara groundnut		0.44		21.61	0.61	Sirivongpaisal (2008)
Rice	0.20	0.03	9.36		0.42	Arns et al. (2015)
Yam	0.12		10.20	24.60		Gunaratne and Hoover (2002)
Cassava	0.11	0.20	13.50	19.80	0.30	Gunaratne and Hoover (2002), Alcazar-Alay and Meireles (2015)
Sweet potato				28.90		Tan et al. (2006)
Wild sorghum		0.80		23.70–27.60	2.30	Alcazar-Alay and Meireles (2015)
Wild barley		0.70–1.20		19.00–22.10	0.20–0.40	Alcazar-Alay and Meireles (2015)

### Granule characteristics of cereals, legumes and tuber starch

Starch granules comprises alternating amorphous and semi-crystalline rings. Granule morphology, size and surface properties, however, plays a great role in food and industrial utilization of starches. The functional properties of starch depend on the composition of the molecular structure of AM and AP as well as their arrangement in starch granules and this confers vital effects in food formulations (Madruga et al. 2014). Starches from cereals, legumes and tubers vary in their granular characteristics of shape, size and distribution (Table 2). The differences in size of the starch granules (1–100 μm in diameter), shape (round, lenticular, polygonal), size distribution (unimodal or bimodal), association as individual (simple) or granule clusters (compound) and composition, reveals the botanical source of the starch (Buleon et al. 1998; Fredriksson et al. 1998; Tester and Karkalas 2002; Zobel and Stephen 1996). The level of analytical sophistication used in understanding the structure of starch and how it describes the starch’s functionality has been revealed from several works with environmental factors also leading to observed variations in starch granule dimensions, size and distribution (Tester et al. 2004). Physical, chemical and enzymatic modification of starches can also bring about improved characteristics and alterations in the starch granules. The structure of starches can be described in terms of physicochemical properties of the molecules, compositional variation, interactions at the molecular level, molecular architecture and the macro level of the whole granule itself (Tester et al. 2004). It can therefore be deduced that the shape, size and distribution of a starch granule can be determined by the plant source in which the starch was extracted.

**Table 2:** Granule shape, size and distribution of cereals, legumes and tuber starch

Starch source	Type	Shape	Distribution	Size (μm)
Barley	Cereal	Lenticular (A-type), spherical (B-type)	Bimodal	15–25, 2–5
Maize (waxy and normal)	Cereal	Spherical/polyhedral	Unimodal	2–30
Amylomaize	Cereal	Irregular	Unimodal	2–30
Millet	Cereal	Polyhedral	Unimodal	4–12
Oat	Cereal	Polyhedral	Unimodal	3–10 (single), 80 (compound)
Pea	Legume	Rentiform (single)	Unimodal	5–10
Potato	Tuber	Lenticular	Unimodal	5–100
Rice	Cereal	Polyhedral	Unimodal	3–8 (single), 150 (compound)
Rye	Cereal	Lenticular (A-type), spherical (B-type)	Bimodal	10–40, 5–10
Sorghum	Cereal	Spherical	Unimodal	5-20
Tapioca	Tuber	Spherical/Lenticular	Unimodal	5–45
Triticale	Cereal	Spherical	Unimodal	1–30
Sago	Cereal	Oval	Unimodal	20–40
Wheat	Cereal	Lenticular (A-type), spherical (B-type)	Bimodal	15–25, 2–10

Source: Tester et al. (2004)

## Hydrothermal modification of starch

Hydrothermal modifications have been used in altering native starches in order to improve the physicochemical properties of the previously unmodified starches. Modification by hydrothermal means are carried out with a view to address and broaden the range of industrial application of starches (Zavareze et al. 2010). Two primary types of hydrothermal treatments: annealing and HMT are commonly employed in the physical modification of starch. These two modification techniques hold advantages over other forms of starch modification in that they result in starches with modified properties without rupturing the starch granule (Adebowale et al. 2009). Annealing is a form of hydrothermal technique that involves treating the starch with excess levels of moisture, whereas HMT involves the addition of restricted levels of moisture for the treatment of the starch (Adebowale et al. 2005a). Numerous studies have been carried out on the influence of HMT and annealing on starches from different plant sources: finger millet (Adebowale et al. 2005a), bambara groundnut (Adebowale and Lawal 2002), mucuna bean (Adebowale and Lawal 2003), sorghum (Adebowale et al. 2005b), pea, lentil, navy bean (Chung et al. 2010), corn, pea, lentil (Chung et al. 2009), potato (Vermeylen et al. 2006), rice (Hormdok and Noomhorm 2007) and wheat (Lan et al. 2008). Most findings on the studies conducted, revealed that both annealing and HMT executes structural changes in the amorphous and crystalline regions in the starches, thus having marked effects on granular swelling, functional, thermal properties, molecular, crystalline structure and susceptibility towards enzyme and acid (Chung et al. 2010). Annealing and HMT are two hydrothermal methods that have also been applied to modify starch digestibility (Chung et al. 2009).

### Heat-moisture treatment

Heat-moisture treatment changes the physical and functional properties of starch with no alteration to its molecular composition (Hoover 2010). Heat-moisture treatment is an important physical method of improving the weak functional properties of native starch needed for diverse food applications (Li et al. 2011). Gunaratne and Hoover (2002) defined HMT as a hydrothermal modification in low moisture contents of less than 35% w/w and the exposure of starch granules to temperatures higher than the glass transition temperature (*T*_*g*_), but lower than the onset temperature (*T*_*o*_) of gelatinization for a specified time. Kweon et al. (2000) further described HMT as a method of exposing starch to heat at elevated temperatures accompanied by starch granules incubation in changing moisture contents (18–27%) for a duration of up to 16 h at a temperature of 110 °C. Changes caused by HMT have been studied in maize (Jiranuntakul et al. 2011), pea and lentil (Chung et al. 2009), rice (Khunae et al. 2007), wheat (Majzoobi et al. 2017), cocoyam (Lawal 2005), barley and rye (Hoover 2010), sorghum (Adebowale et al. 2005b; Olayinka et al. 2008), wheat (Stute 1992) and yam (Tattiyakul et al. 2006). The implication of HMT process on starches has been linked to the moisture content upon heat treatment as well as the botanical source of the starch (Hoover and Manuel 1996; Liu et al. 2016).

**Fig. 2:**
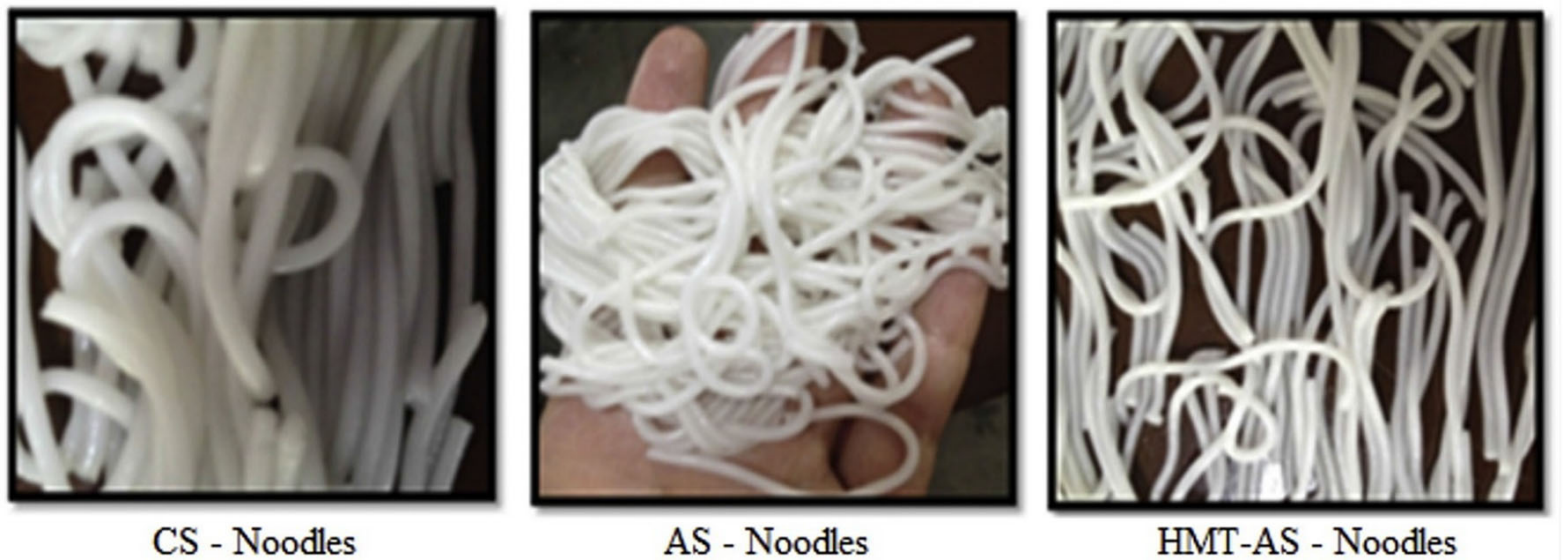
Noodles produced from native (corn and grain amaranth) and heat-moisture treatment starches. CS—Noodles = corn starch noodles; AS—Noodles = amaranth starch noodles; HMT-AS—Noodles = heat-moisture treated amaranth starch noodles. Source: Chandla et al. (2017)

The use of HMT in starch modification, changes several starch characteristics using easy and environmentally safe processes as presently demanded by consumers, in the production of quality food products (Olayinka et al. 2008). Such products include noodles (Fig. 2), baked food products and pastes. Heat-moisture treatment can enhance starch thermal stability and lower the point of set-back (Adebowale et al. 2005a). Jacobs et al. (1997) and Ahn et al. (2013) further reported that HMT changes the crystallographic pattern of starch granules, thus inducing the transformation of a fraction of amorphous AM to its crystalline form. Modified starches from crops such as sweet potato, cassava, arrow root, potato and mung bean are considered to have better swelling power and show type A- and C- X-ray diffraction pattern. Type A- diffraction pattern is the crystalline arrangement of the external AP chains, while C-type crystalline polymorph is formed from a mixture of the A- and B-types (Zhu 2015). Starch with the type C- diffraction pattern forms a unique raw material for products such as starch noodles as they impart translucent and fine threads with better tensile power and less cooking loss when subjected to extended cooking (Collado et al. 2001). The reduction in granular swelling and AM leaching as well as the enhanced heat and shear stability that take place during HMT are all advantageous for noodle manufacture (Hormdok and Noomhorm 2007). Hence, heat-moisture treated starch holds essential qualities required in food industries. Furthermore, the potential of increasing the functional properties of flour from tuber crops such as sweet potato using physical modification has been noted and this could assist in generating new applications of the flour (Ahn et al. 2013). Most heat treatments modify flavour and overall acceptability of the resultant food product.

### Annealing

Annealing of starch involves physical modification of starch granules under conditions of heat and excess water (less than 60% water, w/w) or at intermediate water content (≥ 40% water, w/w). During the annealing process, starch granules are kept at temperatures above their *T*_*g*_ but way below their gelatinization temperature for a specified period (Tester and Debon 2000). Annealing enhances the thermal stability and reduces the degree of set-back of starches (Adebowale and Lawal 2002). Starches obtained through annealing have found application in canned and frozen food products as well as in the preparation of rice noodles processed from long-grain rice that has undergone long storage period. While long storage period is known to limit swelling of starch granules, it is reported to enhance starch paste and gel. The reduction in granular swelling and AM leaching, as well as the improvement of heat and shear stability that occurs during annealing forms part of the desired characteristics for noodle production (Zhou et al. 2003). Accordingly, Hormdok and Noomhorm (2007) explored the use of annealed and native rice starches as alternative ingredients for the processing of noodles of improved quality. The authors in their study, compared textural qualities of chewiness, adhesiveness and tensile strength of rice noodles obtained from annealed rice starch with that of commercial noodles and found them comparable.

**Fig. 3:**
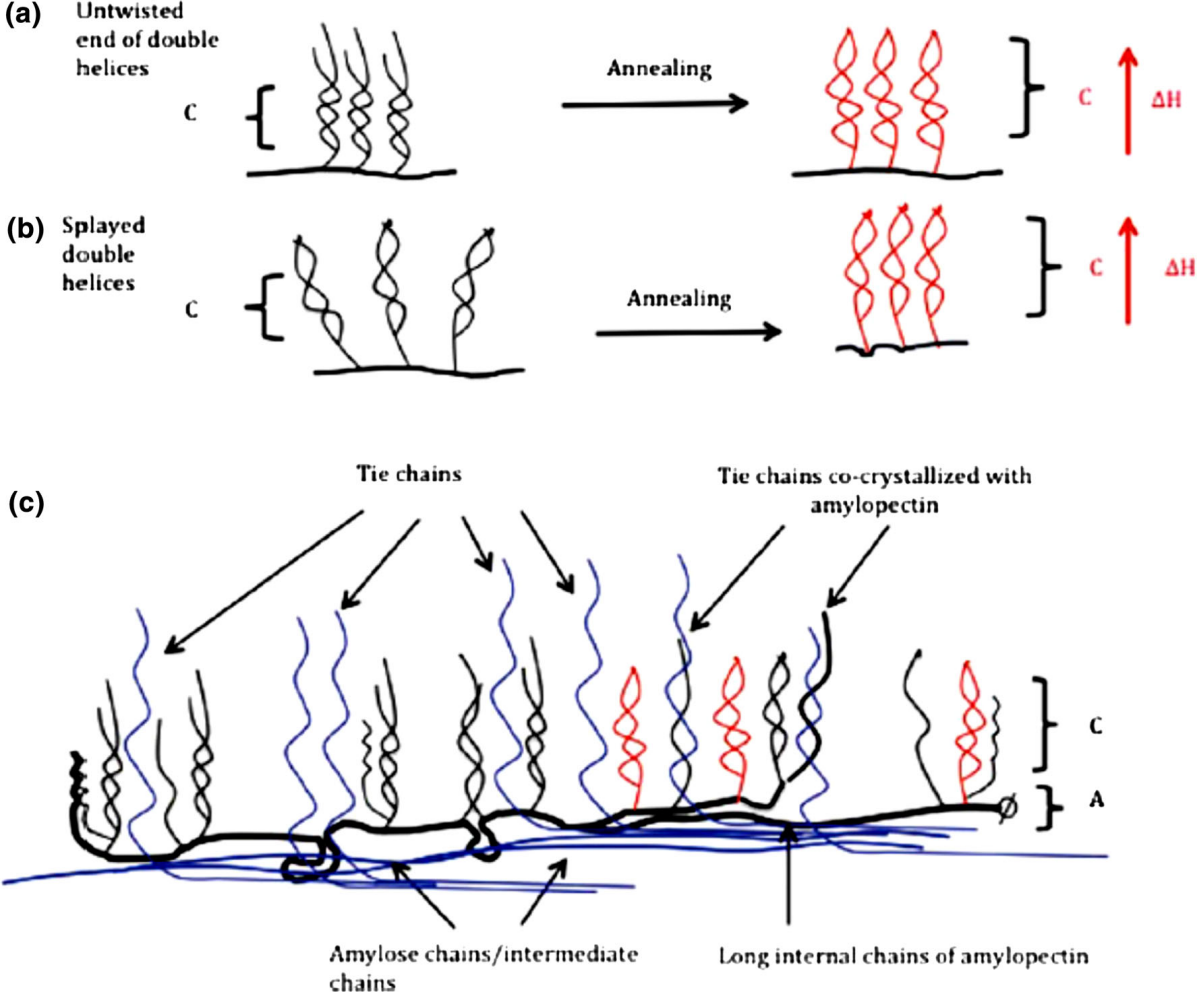
Structural improvement on annealing. **a** lengthening of the double helices by twisting of the ends of the chains; **b** improved parallel packing of double helices; **c** co-crystallization of tie chains and amylopectin. Upright arrows in (**a**) and (**b**) depicts that annealing increases the crystalline thickness and thus the enthalpy of gelatinization. *Source*: Vamadevan et al. (2014)

Annealing modification technique is further associated with increase in granule structure stability, formation of perfect crystalline, starch chain connections in amorphous and crystalline domains of granule, as well as in the emergence of double helices in starch granule (Fig. 3). It also improves some gelatinization parameters, but reduces the gelatinization temperature range, narrows swelling of granules and lowers the leaching of AM (Tester and Debon 2000; Hoover and Manuel, 1996; Hoover and Vasanthan 1994; Jacobs and Delcour 1998; Waduge et al. 2006). Based on the source of the extracted starch, annealing brings about an influence on the granular structure as well as the functional and thermal properties of the different starches (Adebowale et al. 2005b, 2009; Chung et al. 2009). Noda et al. (1998) established through a study on annealed sweet potato and buckwheat starches by differential scanning calorimetry (DSC) that gelatinization parameters are somewhat impacted by the molecular architecture of AP, but not by the AM to AP ratio. The authors opined that lower gelatinization temperatures depict the presence of numerous short AP chains in the starch. These short chains can form stable double helices that would need less energy to untangle and melt during gelatinization and ultimately result in decreased gelatinization temperatures (Chung et al. 2009; Srichuwong and Jane 2007).

Adebowale et al. (2005b) discovered that annealing of red sorghum starch augmented its water absorption capacity (WAC) which is believed to portray that the amorphous region may expand slightly and hydrogen bonds between the amorphous and crystalline regions could be broken. The authors further observed an increased oil absorption capacity (OAC), reflecting the lipophilic nature of the outer covering formed in the sorghum starch granule exterior during annealing. The hydrothermal modification method has also been associated with narrowing of the level of protein in lima beans (Bentacur and Chel 2001) and yam bean (Adebowale et al. 2009). Adebowale et al. (2009) further observed yam starch granules under scanning electron microscopy and noted a surface indentation and emergence of groves in the central region of the granules alongside folding and development of doughnut-like appearance in yam bean starch granules. This is believed to be caused by lengthy treatment period of the starch in excess moisture during the modification process.

## Effect of heat-moisture treatment on some properties of starch from different botanical sources

### Influence of heat-moisture treatment on composition of starch

Recent studies on the effect of HMT on starches include studies on amylomaize, maize, dull waxy maize, waxy maize (Chung et al. 2009; Jiranuntakul et al. 2011) and rice starches (Arns et al. 2015; Anderson and Guraya 2005; Khunae et al. 2007). In the works of Gunaratne and Hoover (2002), it was observed that HMT changed the X-ray diffraction pattern of potato and true yam starches from the B-type to “A + B” type starches. However, HMT did not have any effect on the X-ray diffraction pattern of taro, cassava and new cocoyam starch. Arns et al. (2015) showed that HMT did not significantly affect the ash and level of moisture in buckwheat starch but improved the AM content of the starch (Table 3). This is possibly due to the associations between AM–AM and AP–AP chains (Sarkar 2015). These findings were also supported by Olayinka et al. (2008). Ambigaipalan et al. (2014) postulated that HMT had no effect on the AP chain length distribution of pulse starches, though observed increases in crystallinity and gelatinization temperature were noticed in all starches.

**Table 3:** Influence of heat-moisture treatment on the chemical composition of tuber, cereal and legume starch

Starch source	Starch conditions	Treatment conditions	Protein (%)	Ash (%)	Moisture (%)	Lipid (%)	Amylose (%)	Carbohydrate (%)
Rice^a^	Native	120 °C for 30 min	0.42	0.20	9.36	0.03		
	HMT	13% moisture	0.80	0.14	9.45	0.07		
Buck^b^ wheat	Native	110 °C for 4 h		0.16	10.19		22.80	
	HMT	25% moisture		0.16	10.83		25.70	
Yam bean^c^	Native	100 °C for 16 h	1.70	0.80	8.20	0.70	35.20	86.50
	HMT	18% moisture	1.10	0.70	6.70	0.40	34.20	82.50
Sago^d^	Native	110 °C for 2–6 h	0.12	1.56	15.80	0.80	23.97	81.73
	HMT	28% moisture	0.24	0.79	17.27	0.84	24.22	80.86
Potato^e^	Native	120 °C for 1 h	0.40	0.38	10.62	0.20	27.60	
	HMT	20–25% moisture	0.33	0.34	12.19	0.17	25.72	

*HMT*  heat-moisture treatment

^a^Arns et al. (2015), ^b^Sarkar (2015), ^c^Adebowale et al. (2009), ^d^Liestianty et al. (2016), ^e^Nadir et al. (2015)

### Influence of heat-moisture treatment on resistant starch content

Application of HMT in starch from various plant sources have shown tremendous effect in positively influencing the concentration of resistant starch (RS). Literatures on the effect of HMT on RS content of starches from various sources, have presented increases in the RS concentration of these starch. Li et al. (2011), noted that at HMT of 20% moisture content, the RS concentration of starch from different sources were about 2–4 times greater than that of the control. Accordingly, it has been reported that a major factor for the increase in RS content of HMT modified starch is the moisture content. As seen in the works of Kurakake et al. (1997), RS was higher in HMT with higher moisture content concentration than in samples having less moisture content. This can majorly be attributed to the fact that water creates hydrogen bonds between molecular chains within the starch granules. Kim et al. (2017) observed that RS increased with increase in heating temperature and time. Increase in RS could be due to improved starch chain mobility as a result of increased heating temperature and time, thus leading to decreased enzyme susceptibility. As stated by Chung et al. (2009), increase in RS observed in maize, pea and lentil starches can be linked to the interactions formed between AM–AM chains as well as resisted disruption during cooking, thus hindering the accessibility of starch chains to hydrolysing enzymes. The authors further observed that increasing the HMT temperature and time will further lead to a decrease in digestibility of starch from the rice flour.

### Influence of heat-moisture treatment on the functional properties of starch from different botanical sources

The potential of increasing the functional properties of flour from tuber crops such as sweet potato using physical modification has been noted and this could assist in generating new applications of the flour (Ahn et al. 2013). Heat-moisture treatment induces alterations in the crystalline and amorphous regions in starch grains thus leading to the modification of some properties of the starch. This process leads to the development of double helices, thus limiting starch swelling and solubility (Lawal 2005). Jyothi et al. (2010) postulated that the HMT process also increases other associations among AM–AM and AM-AP chains. This results in an impenetrable particle structure that is accountable for the reduction in swelling power. The arrangement of AM-lipid complexes inside the starch particles has also been associated with the reduction of the swelling capacity because AM restrains granule swelling in surroundings where AM-lipid complexes are created (Noda et al. 1998). Zavareze and Dias (2011) stated in their work that the structural alteration in the starch granules upon HMT may result in the decline of the swelling power and starch solubility.

Adebowale et al. (2005b) in their study on the effect of HMT and annealing on the physicochemical properties of red sorghum starch, reported that different starches swell in diverse ways, thus demonstrating variations in the molecular organization inside the granules. The authors further suggested an association between temperature and swelling power thereby showing an improvement in the swelling power of starch upon increase in temperature. Klein et al. (2013) investigated the influence of HMT on the solubility of cassava and rice starches and discovered that native starches had higher solubility values compared to the heat-moisture treated starches. Heat-moisture treatment decreases the solubility of starch due to interactions involving AM–AM and AP–AP chains (Olayinka et al. 2008; Sarkar 2015). The use of HMT allows the AM molecules positioned in the bulk amorphous regions to interrelate with the branched section of AP in the crystalline regions (Hoover and Manuel 1996). Starch solubility is equally influenced by AM leaching as it dissociates and diffuses out of the starch granule during swelling.

Sarkar (2015) reported an expansion in WAC of starches by HMT. This implies that hydrophilic tendencies in starch increases with an increase in the levels of moisture treatment. Malik and Saxena (2016) stated that WAC indicates the degree of water absorption and association of the molecules with starch granules. Modification by HMT showed a considerable increase in WAC of buckwheat starch. Nadir et al. (2015) in their work also modified buckwheat starch through HMT and noted an increase in the WAC of the samples. In another study conducted on arrowroot starch, HMT hardly changed the water absorption index and water solubility index. However, it reduced swelling power and solubility of the starch at 90 °C. This suggests that HMT led to restructuring of starch chains which is suitable for a repositioning of bonds of the starch chains (Chung et al. 2009; Gunaratne and Hoover 2002). Malik and Saxena (2016), reported a decrease in OAC of heat-moisture treated buckwheat starch. In their findings, OAC was seen to range from 82.16 to 91.84% upon modification by HMT. Furthermore, the authors established that the lowest OAC was observed at increased temperatures and moisture concentration.

Collar and Armero (2018a) observed that HMT affects the viscoelasticity and functionality of blended doughs from different combinations of wheat and non-wheat flours. The works of the author showed studies highlighting the importance water and HMT of compositional flours in obtaining a reinforced dough structure with restored viscoelasticity. As stated in the works of Collar and Armero (2018b), gluten proteins were found to be responsible for the exclusive viscoelastic properties of wheat dough. Addition of water and homogenisation led to the unfolding and construction of a transient network consisting of mostly disulphide bonds, favourable arrangement of hydrogen bonds, hydrophobic interactions as well as entanglements. Application of thermal treatments to the conformation of the proteins also results in the modification of the dough formation process. Heat-moisture treatment was seen to lead to decrease in protein solubility together with gluten protein denaturation as well as aggregation in wheat systems. The study further showed that protein layers in association with increased hydrophobicity, reduced the swelling of HMT starch granules in wheat flour (Chen et al. 2015; Collar and Armero 2018b). Heat-moisture treatment can lead to the hydrophobicity of granule’s surface thus conferring on starch granules, more rigidity and resistance to the quick heating through alteration of swelling behaviour (McCann et al. 2013; Collar and Armero 2018b).

### Influence of heat-moisture treatment on thermal properties and retrogradation of starch from different botanical sources

Thermal properties of starches are affected by the application of HMT depending on the botanical source of the starch being investigated. Heat-moisture treatment (irrespective of treatment time used) has been observed to significantly affect modified starches by influencing its gelatinization and enthalpy change. Cooke and Gidley (1992) reported that enthalpy depicts the breaking of double helix array rather than depletion of crystallinity throughout gelatinization. Pepe et al. (2015) observed that there was a decline in the enthalpy change of arrowroot starch upon HMT. The study showed that the treatment possibly leads to an unwinding of double helices. Furthermore, use of HMT was observed to increase the *T*_*o*_, peak (*T*_*p*_) and concluding temperature (*T*_*c*_) of pea and lentil starches with increase in temperature (Chung et al. 2010). Hoover and Vasanthan (1994) attributed these observed variations to changes within the composition of starch granules and the interface between branched fragments of AP in the crystalline parts. Such interactions reduce the movement of the AP chains, resulting in an amplified *T*_*o*_, *T*_*p*_ and *T*_*c*_. A major increase in gelatinization properties has been reported for most treated rice starches with each gelatinization property amplified with a boost in the levels of moisture during treatments (Table 4). Lim et al. (2001), showed that increase in gelatinization occurred as a result of alteration of the inter-crystalline parts into amorphous parts during the HMT process.

**Table 4:** Thermal properties of native and heat-moisture treated tuber, cereal and legume starch

Starch source	Starch conditions	Treatment conditions	*T*_*o*_ (°C)	*T*_*p*_ (°C)	*T*_*c*_ (°C)	ΔH (J/g)	References
Finger millet	Native	100 °C for 16 h	68.16	70.62	73.79	9.64	Adebowale et al. (2005a)
	HMT	20% moisture	72.48	81.77	85.08	5.73	
Rice	Native	100 °C for 16 h	69.20	73.10	77.90	14.90	Liestianty et al. (2016)
	HMT	20% moisture	72.20	75.90	82.70	16.10	
Yam bean	Native	110 °C for 16 h	70.10	75.06	80.96	1.28	Puncha-arnon and Uttapap (2013)
	HMT	27% moisture	75.30	81.02	86.50	0.11	
Pea	Native	50 °C for 24 h	60.40	67.20	82.30	12.00	Chung et al. (2010)
	HMT	70% moisture	63.60	74.00	89.20	11.40	
Lentil	Native	50 °C for 24 h	64.00	70.00	84.90	13.50	Gunaratne and Hoover (2002)
	HMT	70% moisture	67.20	74.40	91.10	13.10	
Cassava	Native	100 °C for 10 h	63.00	71.50	81.50	12.30	Gunaratne and Hoover (2002)
	HMT	30% moisture	66.40	79.10	87.00	11.70	
Potato	Native	100 °C for 10 h	59.60	66.30	76.00	16.30	Gunaratne and Hoover (2002)
	HMT	30% moisture	61.20	75.60	86.50	11.50	
Corn	Native	100 °C for 16 h	67.30	73.00	82.70	9.30	Ubwa et al. (2012)
	HMT	20% moisture	65.40	71.40	82.10	98.80	

*T*_*o*_ = Onset temperature, *T*_*p*_ = peak temperature, *T*_*c*_ = end set temperature, ΔH = enthalpy change, HMT = heat-moisture treatment

Starch retrogradation is an unstable thermo changeable re-crystallization process which occurs through three successive stages of propagation, nucleation and maturation (Hoover and Vasanthan 1994). Retrogradation process is accompanied by gel firmness, crystallinity, manifestation of a B- X-ray diffraction pattern and turbidity. Retrogradation further occurs because crystallization increases granule rigidity and improves the strengthening of AM matrix (Hoover and Vasanthan 1994; Ambigaipalan et al. 2013). Hoover (1995) showed that retrogradation during HMT on wheat, potato, oat and lentil starches using the DSC revealed a retrogradation endotherm that appeared after 3 days. During storage, the arrangement and cross alliance of dual helices linking AP chains was fierce and occurred more swiftly in HMT starches than in native starches. Hoover (2010) further observed that HMT enhances AM-AP interactions due to an increase in chain mobility, thus resulting in less disruption of hydrogen bonds of the starch during gelatinization. In the DSC studies by Takaya et al. (2002), HMT of maize starch increased the level and rate of retrogradation during storage at 5 °C, while X-ray diffraction studies also showed that retrogradation of maize and potato starches significantly increased during HMT. Furthermore, Adebowale and Lawal (2003) reported during DSC studies on mucuna bean starch that HMT decreased retrogradation in starches stored at 30 °C for two and seven days. It can thus be stated that the conditions of storage and HMT plays a major role in the retrogradation of starches from different plant sources.

### Influence of heat-moisture treatment on the granular orientation of starch from different botanical sources

The influence of HMT on granular orientation of starch has been conducted on starches from several botanical sources such as potato (Vermeylen et al. 2006; Stute 1992; Kawabata et al. 1994), yam (Tattiyakul et al. 2006), cassava (Gunaratne and Hoover 2002), wheat (Liu et al. 2015), maize (Hoover and Vasanthan 1994), rice (Khunae et al. 2007; Zavareze et al. 2012), finger millet (Adebowale et al. 2005a), bambara groundnut (Adebowale and Lawal 2002), mucuna bean (Adebowale and Lawal 2003), mung bean (Li and Gao 2010), canna (Watcharatewinkul et al. 2009) and cocoyam starches (Lawal 2005). As reported by Hoover (2010), most starches granular orientation is not usually affected by modification through HMT. However, at temperatures exceeding 110 °C, the development of hollow regions as well as the fading of birefringence at the granule center were noted in potato (Vermeylen et al. 2006; Kawabata et al. 1994) and maize (Kawabata et al. 1994) starches. Despite these changes in starch, the granule periphery remained highly birefringent even after modification by HMT (Vermeylen et al. 2006). Khunae et al. (2007) observed rice starch granules through a scanning electron microscopy and reported that the granules of the unmodified starch were tiny and had likely angular and polyhedral shapes with a smooth surface. It was also observed that the application of HMT did not alter the appearance of the granules and no damage was induced by the application of HMT. Similar findings were reported for heat-moisture treated bambara groundnut, mucuna bean, canna, cocoyam and finger millet starches (Adebowale et al. 2005a; Adebowale and Lawal 2002, 2003; Lawal 2005; Watcharatewinkul et al. 2009). In mung bean starch however, the granules were reported to be rounded, kidney and oval irregularly shaped. Their findings further showed that smaller starch granules were rounded, while the larger starch granules seemed elliptical. Surface of native starch granules were seen to be smooth without fissures while the shape of granules was also not altered during HMT (Li and Gao 2010). Zhang et al. (2010) showed that morphology of starch granules during application of HMT is moisture dependent. Heat-moisture treatment changes the crystallographic pattern of starch granules, thus inducing the transformation of a fraction of amorphous AM to the crystalline form (Jacobs et al. 1997; Ahn et al. 2013). A study conducted by Zavareze et al. (2010) showed that HMT decreases the relative crystallinity with an increase in the moisture of HMT starches. Zavareze et al. (2012) further observed that rice starches treated with higher moisture content of 20 and 25% were greatly altered in their granular orientation with signs of disintegration in comparison to unmodified starches.

### Influence of heat-moisture treatment on the birefringence patterns of starch granules from different botanical sources

As postulated by Li et al. (2011), birefringence represents a symbol of the mean radial orientation of helical structures of starch granules. Accordingly, occurrence of birefringence in starch granules: from the center to the periphery of the granule, is dependent on the starch source and moisture content concentration during heat treatment. Observed loss of birefringence at the periphery of starch granules can be attributed to increased moisture leading to the disorientation of chains. However, increasing the temperature during HMT treatment could result in reduced birefringence at the center and periphery of starch granules (Chung et al. 2009). Chung et al. (2010) observed that birefringence decreased in all starches upon HMT. Birefringence upon HMT was observed to disappear at the center of the granules only to be retained in the periphery of the granules of pea and navy bean starch. However, there was no change in birefringence in starches examined upon treatment with ANN.

In a study conducted by Ambigaipalan et al. (2011), it was observed that granules of starches obtained from black bean and pinto bean showed strong birefringence patterns while granules from starches of faba bean showed both strong and weak birefringence patterns. Weak birefringence patterns of granules are indicative properties of disorganized AP double helices within the crystalline lamella of the granules. However, in pulses, HMT was observed to show a decrease in the birefringence intensity of the granules. The decrease in intensity could be attributed to a change in radial orientation of AP crystallites as a result of increased AP chain flexibility (Ambigaipalan et al. 2014). Furthermore, at increased temperatures of 120 °C and above, HMT have been reported to lead to a decrease in birefringence around the hilum region in potato (Vamadevan et al. 2011; Vermeylen et al. 2006) and pulse (Chung et al. 2010) starches. Vermeylen et al. (2006), Chung et al. (2010), Vamadevan et al. (2011) and (Vamadevan et al. 2014), further postulated that observed increase in starch chain flexibility that accompanies an increase in HMT temperature, interrupts the radial orientation of AP double helices that are slackly organized around the hilum.

## Uses and application of heat-moisture treatment

### Noodle manufacture

Application of HMT in starch noodles include, raw starch noodles, plain boiled as well as sautéed noodles prepared from HMT using starch from different plant sources. Sweet potato starch has been shown to be unacceptable for the production of bihon type noodles due to their sticky nature and adherence to each other when dried and on rehydration (Collado et al. 2001). However, when 50% heat-moisture treated sweet potato starch and 50% maize starch were used in production of noodles, the noodles obtained was shown to be close to that of commercial bihon type noodles with regards to colour and texture. Noodles have also been produced from sweet potato starch using the HMT technique (Tsakama et al. 2013). Sago starch has also been used in the manufacture of noodles prepared from HMT (25% moisture, at 110 °C for 16 h). The noodles obtained from the sago starch showed increased toughness and flexibility as well as decreased adhesiveness in comparison to those prepared from native starch. Lower cooking loss, rehydration weight and increased cooking time were also reported (Purwani et al. 2006). Collado et al. (2001) reported that sensory evaluation of sautéed noodles resulted in 100% heat-moisture treated sweet potato starch noodles, which was most preferred due to their distinct flavor and chewiness. Thus, application of HMT in noodles produced from different starch sources improves not only the quality, but acceptability of the final product. Table 5 further shows the sensory evaluation of bihon type noodles of HMT starch from four varieties of sweet potato.

**Table 5:** Sensory evaluation of bihon type noodles of heat-moisture treated starch from four varieties of sweet potato

Sensory properties	White sweet potato HMTS	Yellow sweet potato HMTS	Orange sweet potato HMTS	Purple sweet potato HMTS
Taste	3.08 ± 0.17	3.13 ± 0.15	2.88 ± 0.17	3.07 ± 0.21
Colour	3.00 ± 0.19	3.13 ± 0.19	3.07 ± 0.08	2.89 ± 0.07
Texture	3.10 ± 0.11	3.08 ± 0.07	2.98 ± 0.10	3.09 ± 0.17

*HMTS* heat-moisture treated starch. *Source*: Lase et al. (2013)

### Baking

Heat-moisture treatment has been applied in the production of cake and bread in order to improve quality and texture of the final product. Miyazaki and Morita (2005) investigated the impact of heat-moisture treated maize starch on the characteristics of dough and bread. The study showed that there was an increase in the size and crumb texture of bread baked with heat-moisture treated maize. Though, the firmness of the crumb baked with heat-moisture treated maize with and without the use of butter was reported to show no marked variation.

### Other processed foods

Application of HMT has been shown to enhance thermal stability, resistance to shear and acid stability in most starches. Therefore, HMT is used as an environmentally friendly alternative to chemical modification in canned foods, dressings, batter products and confections (Hoover 2010). Collado and Corke (1999) reported in their study that heat-moisture treated starch obtained from cassava exhibited greater freeze–thaw stability and can be incorporated in pie fillers for improved organoleptic characteristics. This modification has also been utilized towards improving the amount of RS while maintaining crumb structure. Heat-moisture treated starches have found application and can also be advantageous in the canning and frozen food industries (Zavareze et al. 2010).

### Health benefit in diets

The HMT starches due to increased RS have beneficial nutritional properties as they lead to a lowered digestibility in the human body (Arns et al. 2015). Food produce that contains RS: gradual digestion and absorption of carbohydrates, are beneficial for the management of diabetes and obesity by decreasing raised blood sugar levels (Anderson and Guraya 2005). Other health benefits of RS are prevention of colon cancer, hypoglycemia, growth of probiotic microorganism and inhibition of fat metabolism (Sajilata et al. 2006; Brumovsky and Thompson 2001; Sang and Seib 2006). Brumovsky and Thompson (2001) investigated the impact of HMT on RS formation in normal and high amylose (ae VII) maize starches. The findings established that upon partial acid hydrolysis, preferential attacks on the amorphous portions of the starch granules will provide possible freedom for chain ends to form double helices, thus enabling HMT to allow mobility of the chains that will lead to the formation of highly ordered structures which hinders the activity of *α*-amylase. Li et al. (2011) showed an increase in RS content in mung bean starch upon HMT at moisture levels of 15 to 35%. An increase in RS content will result in a compact structure within the starch granules that is more resistant to enzymatic hydrolysis. Use of HMT therefore has a potential in providing a cost-effective technique for the manufacture of high-quality starches required for health promoting applications (Jacobasch et al. 2006).

## Conclusion

As studies on more cheap ways of obtaining less chemically and more organically modified food products are being conducted, the knowledge of starch composition and its functionality in utilized and underutilized crops for industrial use is highly essential. Native starches are limited in use due to inherent characteristics such as cohesive texture, heat and shear sensitivity, lack of clarity, opacity, low viscosity and precipitation during storage. Though showing negative influence on the birefringence and crystallographic patterns of the starch granules, HMT was observed to positively affect the nutritional (resistant starch) as well as the functional properties of the modified starches. Modification of starch through HMT therefore, results in improved quality starch readily available for use and application by food processors and industries. However, further studies are needed on the optimization of the HMT temperature times as well as its effect on other nutritional properties of the starch.
